# Clinique of Prof. Davis, in the Medical Department of Lind University

**Published:** 1860-02

**Authors:** 


CLINIQUE OF PROF. DAVIS, in the Medical Department of Lind University. Saturday Afternoon.

Case 1st. *Acute General Dropsy.* The remarks on this case were substantially as follows:—The patient, a native of Ireland, aged about 38 years, was engaged some three weeks since in labor on the prairie, where he got thoroughly wet by a shower of rain, and slept on the damp ground in a temporary shanty.

This was followed by a fever, accompanied by severe pain in the head and back, with decided scantiness of urine. No regular medical aid was obtained, but the patient says his fever subsided in five or six days, but the pain in the loins continued and his feet and ankles began to swell. This swelling rapidly increased, until at present he his universally œdematous; the lower extremities being very much swollen, and pitting deeply on pressure; also a moderate amount of effusion into the abdominal cavity. His pulse is still more full and frequent than natural; his tongue coated; his urine diminished in quantity, and pale in color; with a moderate degree of pain in his back. Those members of the class who have attended the Mercy Hospital and listened to the comments on a case of ascites, a few days since, will readily recognize this as a case of general dropsy; and consequently arising from some cause capable of altering the relative proportion of the constituents of the blood. One of the most common and important of these causes, is such a pathological condition of the kidneys as allows the albumen of the blood to escape with the urine. As the viscidity of the blood depends much on the albumen, whenever the latter is diminished in quantity by excretion through the kidneys, the whole mass of the blood becomes thinner, and dropsical effusions very generally occur.

Finding by auscultation no evidence of organic disease of the heart, and nothing in the history of the case to indicate chronic ague or other causes of anemia, attention must be turned to the kidneys as a probable seat of disease. The exposure to cold and wet that immediately preceded the attack of sickness in this patient, the pain in the loins, the fever, and the scantiness of urine, which characterized the early part of the sickness, soon followed by œdematous effusions into the cellular tissue, point strongly to the kidneys as the seat of such a degree of inflammatory engorgement as to cause the excretion of albumen. This can only be determined certainly, however, by applying the proper chemical tests to the urine. The patient having brought a specimen of his urine in a vial, it was subjected to the tests of heat and nitric acid, before the class. Both the tests caused an abundant precipitate of albumen. The lecturer observed that we had now demonstrative proof that the action of the kidneys was perverted, and that the amount of albuminous excretion was sufficient to impoverish the blood and explain the appearance of the dropsy. But this did not complete the diagnosis. The question now remains, whether the perverted action of the kidneys, is dependent on a simple hyperæmic or inflammatory condition of their secreting structure, or on that peculiar organic change which is properly denominated albuminuria, Bright's disease, or granular kidney? Practically this is a question of much importance.

On its proper solution depends both the correctness of the prognosis and the success of the treatment; the true granular kidney being to a great extent incurable, while those cases of acute dropsy with albuminous urine, dependent on mere inflammatory congestion of the kidneys, can generally be speedily relieved, especially if brought under treatment in the early stage of its progress. In making the diagnosis on this point, we must rely much on the manner of the attack. If the health of the patient has declined slowly, feeling depressed in mind with various dyspeptic symptoms, and more or less pains in the loins, with a sense of weakness for several months before any signs of œdema appear; if the latter increases very slowly with a corresponding increase of the preceding symptoms, the urine itself being small in quantity and pale in color, it is quite certain that the disease is a granular degeneration or organic disease of the kidneys. On the other hand, if the attack of sickness has come on suddenly with fever, after a sudden exposure to wet or cold, or during the convalescense from some idiopathic or eruptive fever, and the dropsical effusions have been developed with almost equal rapidity, it is more than probable that the disease is simply inflammatory in its nature. The class will readily recognize the patient before them as belonging to the latter class. His previous good health, the severe exposure to wet and cold, the fever and pain in the back that immediately followed, ending in a few days in general œdema of the cellular tissue, are sufficient to show that the present condition of the patient could not depend on a slow change of structure like the true granular degeneration, or Bright's disease. Being fully satisfied that the disease was inflammatory, the lecturer directed six powders, each containing calomel 2 grs., pulv. degitalis leaves 2 grs., and pulv. doveri 5 grs., to be given every four hours; and when all are taken follow them by sufficient rheubarb and cream of tartar to move the bowels briskly. After this he advised one of the same powders to be given each morning, noon and night, until five or six more had been taken; then omit the calomel and substitute in its place 8 grs. of nitrate of potassa.

He also directed that after the operation of the first dose of physic, the patient should use a solution of bi-tartrate of potassa as a drink. This course of treatment was carried out during the succeeding week, and on Saturday the patient returned to the clinique very much improved.

The bowels had been kept soluble, the urine had increased in quantity, and the amount of dropsical effusion had been much reduced. A specimen of the urine was exhibited to the class, and subjected to the action of heat and nitric acid, and although there was still a trace of albumen, its quantity was very much diminished. He was directed an infusion of uva ursi, nigitalis, and nitrate of potassa, to be taken four times a day.

On his return to the clinique the following week, he appeared quite well; his urine was abundant and presented no trace of albumen.

